# Medical and neurobehavioural phenotypes in carriers of X-linked ichthyosis-associated genetic deletions in the UK Biobank

**DOI:** 10.1136/jmedgenet-2019-106676

**Published:** 2020-03-05

**Authors:** Lucija Brcic, Jack FG Underwood, Kimberley M Kendall, Xavier Caseras, George Kirov, William Davies

**Affiliations:** 1 School of Psychology, Cardiff University, Cardiff, UK; 2 MRC Centre for Neuropsychiatric Genetics and Genomics and Division of Psychological Medicine and Clinical Neurosciences, School of Medicine, Cardiff University, Cardiff, UK; 3 Neuroscience and Mental Health Research Institute, Cardiff University, Cardiff, UK

**Keywords:** arrhythmias, copy-number, dermatology, psychiatry, neurosciences

## Abstract

**Background:**

X-linked ichthyosis (XLI) is an uncommon dermatological condition resulting from a deficiency of the enzyme steroid sulfatase (STS), often caused by X-linked deletions spanning *STS*. Some medical comorbidities have been identified in XLI cases, but small samples of relatively young patients has limited this. *STS* is highly expressed in subcortical brain structures, and males with XLI and female deletion carriers appear at increased risk of developmental/mood disorders and associated traits; the neurocognitive basis of these findings has not been examined.

**Methods:**

Using the UK Biobank resource, comprising participants aged 40–69 years recruited from the general UK population, we compared multiple medical/neurobehavioural phenotypes in males (n=86) and females (n=312) carrying genetic deletions spanning *STS* (0.8–2.5 Mb) (cases) to male (n=190 577) and female (n=227 862) non-carrier controls.

**Results:**

We identified an elevated rate of atrial fibrillation/flutter in male deletion carriers (10.5% vs 2.7% in male controls, Benjamini-Hochberg corrected p=0.009), and increased rates of mental distress (p=0.003), irritability (p<0.001) and depressive-anxiety traits (p<0.05) in male deletion carriers relative to male controls completing the Mental Health Questionnaire. While academic attainment was unaffected, male and female deletion carriers exhibited impaired performance on the Fluid Intelligence Test (Cohen’s d≤0.05, corrected p<0.1). Neuroanatomical analysis in female deletion carriers indicated reduced right putamen and left nucleus accumbens volumes (Cohen’s d≤0.26, corrected p<0.1).

**Conclusion:**

Adult males with XLI disease-causing deletions are apparently at increased risk of cardiac arrhythmias and self-reported mood problems; altered basal ganglia structure may underlie altered function and XLI-associated psychiatric/behavioural phenotypes. These results provide information for genetic counselling of deletion-carrying individuals and reinforce the need for multidisciplinary medical care.

## Introduction

X-linked ichthyosis (XLI (OMIM: 308100)) is an uncommon dermatological condition characterised by the presence of large dark-brown scales,^1^ although much milder skin phenotypes have been reported^2^; it occurs due to deficiency of the enzyme steroid sulfatase (STS), encoded by the X-linked *STS* gene (OMIM: 300747). STS cleaves sulfate groups from steroid hormones (eg, dehydroepiandrosterone sulfate (DHEAS)) altering their solubility/activity; the XLI-associated skin phenotype arises from cholesterol sulfate accumulation in the stratum corneum. 80-90% of XLI cases are caused by complete/partial deletion of *STS*, with remaining cases attributable to *STS* point mutation(s); the typical deletion (~1.5 Mb) encompasses *STS* and a small number of adjacent genes.^3^ In ~85% of cases, the mutation is transmitted from unaffected (or mildly affected) carrier mothers, and in the remainder it occurs de novo during parental gametogenesis.^4^

Several extracutaneous comorbidities have been reported in individuals possessing XLI-associated mutations: a) benign corneal opacities affect ~10%–50% of males with XLI and ~25% of female carriers,^1^ b) ~20% of males with XLI have cryptorchidism; an associated risk of testicular germ cell cancer is less clear and supported by a few case reports^1 5^ and c) delayed/prolonged labour affects ~60% of female carriers.^1 6^ Occasional comorbidities include: pyloric hypertrophy, congenital defect of the abdominal wall, acute lymphoblastic leukaemia, bilateral periventricular nodular heterotopia and end-stage renal failure.^1^ Rare, larger genetic deletions which can include *NLGN4X* and *KAL* are associated with a number of neurological conditions such as epilepsy, intellectual disability, autism and anosmia, and contiguous gene syndromes including Rud, Conradi and Kallman syndromes.^1 3^ To date, samples of patients with XLI and carrier females have been relatively small/superficially phenotyped, and larger-scale studies may reveal hitherto unappreciated comorbidities with implications for morbidity/mortality.

*STS* is expressed in the developing and adult human brain (with highest expression in subcortical structures^7 8^) and may therefore influence neurodevelopment. Boys with XLI are at elevated risk of developing attention deficit hyperactivity disorder (ADHD) and motor problems,^3 9 10^ and also potentially early onset psychosis.^11^ We have previously shown that males with XLI exhibit higher rates of inattention, impulsivity (but not motor impulsivity), mood problems, disruptive behaviour and autism-related traits, and are more likely to be diagnosed with developmental conditions (including ADHD/autism) and mood disorders, when compared with males from the general population.^12^ A study of female carriers revealed a comparable suite of behavioural differences from control subjects, together with an increased likelihood of postpartum depression.^6^ The pattern of behavioural phenotypes seen in males with XLI/carrier females overlaps with that seen in STS-deficient mice,^13–17^ and may therefore be largely ascribed to biological effects of STS deficiency rather than ascertainment or socialisation effects. Previous behavioural phenotyping approaches in XLI have been suboptimal: a) survey-based studies did not collect genetic data and so could not objectively confirm or define *STS* deletion carrier status, and these studies may theoretically have elicited responses from more severely affected individuals inflating risk estimates, b) only relatively early age points have been assessed (average age <45 years) and c) studies have had either no control group, or no contemporaneously recruited control group. No study has yet investigated the cognitive or neurobiological basis of behavioural phenotypes in XLI. To address these limitations/knowledge gaps, here we use the power of the UK Biobank, a large, accessible resource comprising extensively phenotyped and genotyped members of the population of the UK,^18^ to compare medical/medication history, objective/self-reported behavioural/mental health measures, cognition and subcortical neuroanatomy between males and females carrying deletions most commonly seen in XLI and sex-matched non-carrier controls.

## Methods

### Participants

Participants were individuals (aged 40–69 years) recruited under UK Biobank informed consent procedures between 2006-2010, for which anonymised genotype/phenotype data were available.^19^

### CNV calling

Anonymised genotype data were downloaded as raw (CEL) files from the UK Biobank website,^19^ stored on a secure Linux server and analysed with UNIX-based commands. Affymetrix Power Tools (APT) software^20^ was used to generate normalised signal intensity data, genotype calls and confidences. This argument incorporated genotypic sex data downloaded from the UK Biobank website, and compared with sex data generated through PennCNV-Affy software.^21^ Approximately 750 000 biallelic markers were analysed through PennCNV-Affy to process cluster plots. Canonical genotype clusters, Log R ratios and B allele frequencies were generated to complete the PennCNV recommended process for CNV calls. Individuals with deletions of 0.8–2.5 Mb spanning *STS* were identified, with calls and coordinates based on the GRCh37/hg19 genome build; this range was selected to reflect the majority of XLI cases, to facilitate robust CNV calling, and to exclude individuals with multiple complex medical issues arising from large contiguous gene deletions. Following QC, CNV data were available for a total of 418 837 individuals.

### Measures

Hospital diagnoses according to the International Statistical Classification of Diseases and Related Health Problems Revision-10 (ICD-10),^22^ self-reported non-cancer illnesses, relevant questions from the Mental Health Questionnaire (MHQ)^23^ and medication history were analysed. Highest levels of academic qualification and key performance measures on seven cognitive tasks (transformed and converted to z-scores as described previously^24^) were also analysed: number of incorrect matches on the Pairs Matching Test (episodic memory), mean time to correctly identify matches in the Reaction Time Task (simple processing speed), total number of correct answers in the 13-question Fluid Intelligence Test (reasoning/problem-solving),^25^ maximum number of digits remembered in the ‘Digit Span’ task (numeric working memory), number of correct substitutions in a Symbol Digit Substitution Test (complex processing speed) and time to complete two variants of the Trail Making Task (visual attention). Brain images were acquired using Siemens Skyra 3T scanners in UK Biobank’s imaging centres in Cheadle and Newcastle, UK using identical acquisition protocols^26^; T1-weighted brain images were processed using automated methods implemented in FreeSurfer^27^ to obtain volumetric estimates for eight right and left subcortical regions.

### Statistics

Data were analysed using SPSS V.25.0 (IBM). As male and female deletion phenotypes could differ in magnitude and/or nature, two comparisons were performed: male deletion carriers versus male non-carriers, and female deletion carriers versus female non-carriers. Across the overall sample for each cognitive/neuroimaging measure, outlying values >2.2 times the IQR below the first quartile, or above the third quartile, were excluded.^28^ Categorical data were analysed using χ^2^ test (continuity-adjusted for 2×2 analyses)/Fisher’s exact test, with ORs and 95% CIs presented as a measure of effect size. Normally distributed data were compared using unpaired t-test. Ordinal/non-normally distributed data, and small datasets (<30 participants), were compared using Mann-Whitney U test, with Cohen’s d presented as a measure of effect size.^29^ For cognitive analyses with sample sizes >30, hierarchical linear regression controlling for age was performed.^24^ Data are presented as median values (with 95% CIs) or mean±SE of the mean. Two-sided p values <0.05 were regarded as nominally significant, with p values <0.1 after Benjamini-Hochberg adjustment^30^ regarded as surviving correction for multiple comparisons.

## Results

### Identification and characterisation of deletion cases

We identified 86 male and 312 female deletion carriers, and 190 577 male and 227 862 female non-carriers, giving deletion rates of ~1/2200 in males and ~1/730 in females. The mean deletion size was 1.60±0.01 Mb from ChrX:6 487 716–8 087 815, encompassing the *PUDP/HDHD1* (OMIM: 306480), *STS*, *VCX* (OMIM: 300229) and *PNPLA4* (OMIM: 300102) genes and the *MIR4767* microRNA. Male deletion subjects did not differ significantly from male controls in terms of age (58.0±0.8 vs 57.1±0.02 years, respectively, t[190 606]=−0.999, p=0.318); the ages of female deletion subjects and female controls were also equivalent (56.3±0.5 vs 56.7±0.02 years, respectively, t[228 024]=0.736, p=0.461).

### ICD-10 diagnoses

Nine ICD-10 unique descriptive codes were present in >2500 males overall and >2.5% of male deletion subjects, of which two were significantly more common in male deletion carriers than in control males (online supplementary table 1): ‘atrial fibrillation/flutter’ (10.5% vs 2.7%, OR 4.2 (95% CI 2.1 to 8.3), p=0.001) and ‘skin of other and unspecified parts of face’ (5.8% vs 1.5%, OR 4.0 (95% CI 1.6 to 9.9), p=0.01); while these p values would not survive stringent multiple comparison testing taking into account all possible ICD-10 codes, they did survive multiple comparison testing across the nine aforementioned codes (corrected p=0.009 and 0.045, respectively). Ten ICD-10 unique descriptive codes were present in >2500 females overall and >2.5% of female deletion subjects, none of which were significantly more common in deletion than control subjects (online supplementary table 2). Diagnosis rates of developmental and mood/anxiety disorders did not differ between male and female deletion and control subjects, although baseline rates of each were very low across groups (<0.5%) (online supplementary tables 3 and 4).

10.1136/jmedgenet-2019-106676.supp1 Supplementary data

### Self-reported non-cancer illnesses

We compared rates of self-reported non-cancer physical/mental illnesses of interest based on the ICD-10 findings above, or previous literature, across groups (online supplementary tables 5 and 6). Compared with male controls, male deletion cases reported a significantly higher prevalence of blistering/desquamating skin disorder (4.7% vs 0.2%, OR 28.6 (95% CI 10.4 to 78.4), corrected p<0.020) and allergy/hypersensitivity/anaphylaxis (4.7% vs 0.5%, OR 9.1 (95% CI 3.3 to 24.9), corrected p=0.020), and a nominally significantly increased rate of heart arrhythmia (3.5% vs 0.6%, OR 5.9 (95% CI 1.9 to 18.6), p=0.016), atrial flutter (1.2% vs 0.0%, OR 31.1 (95% CI 4.3 to 226.6), p=0.032) and eczema/dermatitis (7.0% vs 2.7%, OR 2.7 (95% CI 1.2 to 6.2), p=0.028). Two conditions related to those previously reported in males with XLI were not reported significantly more frequently in male deletion carriers than male controls: ‘undescended testicle’ (0.0% vs 0.1%, p>0.99) and ‘cataract’ (3.5% vs 1.6%, OR 2.3 (95% CI 0.7 to 7.2), p=0.15). No non-cancer illnesses we assessed were reported significantly more frequently in female deletion than female control subjects.

### Mental Health Questionnaire

Twenty-one male deletion and 95 female deletion carriers completed the MHQ, together with 58 855 male controls and 76 439 female controls. Male deletion carriers reported a significantly higher likelihood of having suffered mental distress preventing usual activities compared with male controls (57% vs 26%, OR 3.8 (95% CI 1.6 to 9.0), p=0.003), but not a greater likelihood of having sought or received help for mental distress; female deletion carriers did not differ significantly from female controls on these measures (table 1). Mental health diagnoses reported in at least one male or female deletion carrier did not differ in frequency between deletion carriers and controls (table 1). No male or female deletion carriers reported having been diagnosed with any of the following conditions: schizophrenia/other psychotic disorder, personality disorder, mania/bipolar disorder, bulimia nervosa, psychological overeating/binge eating, autism spectrum conditions, anorexia nervosa, agoraphobia or ADHD, and none of these conditions differed in frequency between deletion carriers and control subjects (p>0.99).

**Table 1:** Frequency of mental distress, help-seeking behaviour and psychiatric illness in male and female deletion carriers and controls completing the mental health questionnaire

Measure	Male control	Male deletion	Statistical analysis	Female control	Female deletion	Statistical analysis
Ever suffered mental distress preventing usual activities? (Yes/No)	15 068 (26%)/42 882 (74%)	12 (57%)/9 (43%)	χ^2^_[1]_=9.02, p=0.003	29 250/46 323	30/63	χ^2^_[1]_=1.37, p=0.242
Ever sought or received help for mental distress? (Yes/No)	17 522/41 137	7/14	χ^2^_[1]_=0.01, p=0.914	35 122/41 035	39/56	χ^2^_[1]_=0.786, p=0.375
Social anxiety or social phobia (Yes/No)	756/58 099	0/21	P>0.99	892/75 547	1/94	P>0.99
Phobia (other than social or agoraphobia) (Yes/No)	576/58 279	1/20	P=0.187	1256/75 183	2/93	P=0.671
Panic attacks (Yes/No)	2266/56 589	1/20	P=0.562	5197/71 242	6/89	χ^2^_[1]_=0.000, p>0.99
Obsessive compulsive disorder (Yes/No)	329/58 526	1/20	P=0.111	508/75 931	0/95	P>0.99
Depression (Yes/No)	9188/49 667	6/15	P=0.125	19587/56 852	24/71	χ^2^_[1]_=0.000, p>0.99
Anxiety, nerves or generalised anxiety disorder (Yes/No)	6287/52 568	3/18	P=0.486	12889/63 550	16/79	χ^2^_[1]_=0.000, p>0.99

Responses to the remaining questions of the MHQ are presented in online supplementary tables 7 and 8. Male deletion carriers were more likely than male controls to have experienced ‘recent easy annoyance or irritability’ (p<0.001) or to have ‘ever experienced a period of extreme irritability’ (p=0.031), and female deletion carriers were more likely than female controls to have experienced the former (p=0.018) (d≤0.02). Male deletion carriers were significantly more likely than male controls to have experienced ‘prolonged feelings of sadness or depression’, ‘prolonged loss of interest in normal activities’ (OR 3.9 (95% CI 1.4 to 10.6), p=0.009 and OR 3.5 (95% CI 1.5 to 8.6), p=0.006, respectively) and ‘recent restlessness’ (p=0.008, d=0.012); we found weaker evidence for male deletion carriers being more affected than male controls by recent depression-related traits including: ‘feelings of inadequacy’ (p=0.039), ‘trouble concentrating on things’ (p=0.011), ‘thoughts of suicide and self-harm’ (p=0.024) and ‘lack of interest or pleasure in doing things’ (p=0.015) (d<0.015). There was a nominally significantly increased prevalence of some anxiety-related traits in male deletion carriers relative to male controls, notably ‘ever feeling worried, tense or anxious for most of a month or longer’ (OR 2.8 (95% CI 1.2 to 6.7), p=0.027). Male deletion carriers did not differ from male controls with respect to traumatic event exposure, unusual/psychotic experiences, most aspects of alcohol use, cannabis use or happiness/well-being measures. Female deletion carriers differed from controls on some responses (0.01<p<0.05); notably, the former group were more likely to have experienced a period of mania/excitability (OR 2.5 (95% CI 1.2 to 5.1), p=0.02). Interestingly, both male and female deletion carriers reported consuming less alcohol than controls on a typical drinking day (p=0.013 and p=0.033, respectively, d≤0.02).

### Medications

We found no difference in prescription rates for medications commonly used to treat heart arrhythmias (listed in online supplementary table 9) between male deletion carriers and male controls: male deletion (6/86, 7.0%) versus male controls (16 590/190 577, 8.7%) (χ^2^_[1]_=0.142, p=0.706). Nor did we find any evidence for differential prescription of medications commonly used to treat ADHD-related symptoms (online supplementary table 9): male deletion (0/86, 0%) versus male controls (14/190 577, 0%) (p>0.99) and female deletion (0/312, 0%) versus female controls (11/227 862, 0%) (p>0.99). Finally, we found no evidence that prescription rates of medications used to treat mood symptoms (online supplementary table 9) differed across groups: male deletion (5/86, 5.8%) versus male controls (9419/190 577, 4.9%) (p=0.617) and female deletion (31/312, 9.9%) versus female controls (21 008/227 862, 9.2%) (χ^2^_[1]_=0.115, p=0.735).

### Academic qualifications and cognitive function

Neither male deletion, nor female deletion, carriers differed from their respective controls with respect to highest academic qualification achieved (table 2). Male deletion subjects (57.5±0.8 years) and female deletion subjects (56.3±0.45 years) were compared with a subset of closely age-matched male (55–60 years, mean: 57.7±0.01 years) and female (55–60 years, mean: 57.7±0.01 years) control subjects on the cognitive tests. On average, male deletion carriers performed more poorly than male controls across all tasks, exhibiting significantly slower reaction times in the Reaction Time Test (B=0.217±0.101, β=0.010, p=0.032), and providing significantly fewer correct answers in the Fluid Intelligence Test (B=−0.465±0.190, β=−0.021, p=0.014); however, only the latter difference reached significance after correcting for multiple testing (corrected p=0.098) (table 3). Female deletion carriers only demonstrated significantly different (worse) performance from female controls on the Fluid Intelligence Test (corrected p<0.0063, d=0.054) (table 3).

**Table 2:** Highest academic qualification achieved by male and female deletion carriers and controls

Highest academic qualification	Male control (n=1 80 071)	Male deletion (n=78)	Statistical analysis	Female control (n=2 12 817)	Female deletion (n=290)	Statistical analysis
College/University degree	62 371 (35%)	18 (23%)	P=0.083	67 798 (32%)	84 (29%)	χ^2^_[5]_=3.995, p=0.550
A/AS Levels	19 794 (11%)	13 (17%)		27 229 (13%)	37 (13%)	
O Levels/GCSEs	36 430 (20%)	21 (27%)		55 181 (26%)	75 (26%)	
CSEs	10 424 (6%)	2 (2%)		12 550 (6%)	24 (8%)	
NVQ/HND/HNC	17 684 (10%)	6 (8%)		10 100 (4%)	12 (4%)	
None	33 368 (18%)	18 (23%)		39 959 (19%)	58 (20%)	

**Table 3:** Performance on key measures of seven cognitive tasks by male and female deletion carriers and controls

Cognitive task	Control group	Deletion group	Statistical comparison	Benjamini-Hochberg corrected p value
*Male participants*				
Pairs Matching Test (total number of errors)	−0.11 (−0.11 to −0.11) (n=42 990)	0.25 (−0.11 to 0.54) (n=86)	B=0.202±0.112, β=0.009, p=0.071	0.166
Reaction Time Test	−0.19 (−0.19 to −0.18) (n=42 626)	−0.025 (−0.19 to 0.265) (n=86)	B=0.217±0.101, β=0.010, p=0.032	0.112
Fluid Intelligence Test (total number of correct answers)	0.40 (−0.07 to 0.40) (n=13 072)	−0.07 (−0.55 to 0.40) (n=31)	B=−0.465±0.190, β=−0.021, p=0.014	0.098
Digit Span Test (maximum number of digits remembered)	0.21 (0.21 to 0.21) (n=4195)	0.21 (−0.165 to 0.97) (n=10)	U=18 543.5, p=0.512	0.563
Symbol Digit Substitution Test (number of correct substitutions)	0.05 (0.05 to 0.05) (n=11 664)	−0.35 (−0.838 to 0.345) (n=13)	U=63 851, p=0.323	0.452
Trail Making Test A (time to completion)	−0.19 (−0.22 to −0.17) (n=10 580)	−0.04 (−0.43 to 1.07) (n=14)	U=58 547, p=0.175	0.306
Trail Making Test B (time to completion)	−0.11 (−0.13 to −0.10) (n=10 588)	0.125 (−0.53 to 0.44) (n=14)	U=67 499, p=0.563	0.563
*Female participants*				
Pairs Matching Test (total number of errors)	−0.11 (−0.11 to −0.11) (n=54 151)	0.25 (−0.11 to 0.25) (n=312)	B=0.105±0.057, β=0.008, p=0.067	0.235
Reaction Time Test	0.01 (0.01 to 0.01) (n=53 692)	−0.10 (−0.24 to 0.0495) (n=309)	B=−0.0202±0.052, β=−0.002, p=0.697	0.835
Fluid Intelligence Test (total number of correct answers)	−0.07 (−0.07 to −0.07) (n=16 615) (mean: 0.05±0.0074)	−0.07 (−0.55 to −0.07) (n=103) (mean: −0.31±0.0972)	B=−0.352±0.096, β=−0.028, p<0.001	<0.0063
Digit Span Test (maximum number of digits remembered)	0.21 (0.21 to 0.21) (n=5386)	0.21 (−0.54 to 0.59) (n=36)	B=0.058±0.160, β=0.005, p=0.716	0.835
Symbol Digit Substitution Test (number of correct substitutions)	0.05 (0.05 to 0.05) (n=15 163)	0.15 (−0.25 to 0.54) (n=64)	B=−0.063±0.114, β=−0.004, p=0.581	0.835
Trail Making Test A (time to completion)	−0.02 (−0.04 to 0.00) (n=13 179)	0.015 (−0.28 to 0.29) (n=58)	B=0.020±0.126, β=0.001, p=0.875	0.875
Trail Making Test B (time to completion)	0.01 (−0.01 to 0.03) (n=13 206)	0.08 (−0.17 to 0.385) (n=58)	B=0.123±0.121, β=0.009, p=0.307	0.716

Data are presented as median z-scores with 95% confidence limits.

### Subcortical neuroanatomy

Neuroimaging data were available for only two male deletion carriers hence analysis was limited to a comparison between female deletion carriers (n=14) and a subset of female controls (n=415) matched as closely as possible for handedness, scanning centre, age at scanning (61.2±2.9 vs 61.0±0.04 years, respectively, t[4.0]=−0.060, p=0.955), and intracranial volume (1396±39 vs 1399±4 cm^3^, respectively, t[13.2]=0.074, p=0.942). Four subcortical brain regions were nominally significantly smaller in deletion than control subjects (left and right putamen, right pallidum and left nucleus accumbens); two of these comparisons (right putamen and left nucleus accumbens) survived correction for multiple testing (corrected p=0.072, d=0.254 and d=0.264, respectively), while a third almost did (right pallidum, corrected p=0.101, d=0.232) (table 4).

**Table 4:** Volumes of eight subcortical brain regions (right and left hemisphere) in male and female deletion carriers and controls

Hemisphere	Subcortical region	Volume in control female subjects (cm^3^) (n=415)	Volume in female deletion subjects (cm^3^) (n=14)	Statistical comparison Mann-Whitney U value, p value	Benjamini-Hochberg corrected p value
Left	Lateral ventricle	7.62 (7.08 to 8.06)	9.32 (7.50 to 11.18)	2038, 0.059	0.189
	Thalamus	7.15 (7.05 to 7.25)	7.17 (6.19 to 7.53)	2582.5, 0.480	0.640
	Caudate	3.20 (3.17 to 3.24)	3.15 (2.91 to 3.21)	2347, 0.226	0.452
	Putamen	4.84 (4.78 to 4.90)	4.44 (3.82 to 4.99)	1912, 0.030	0.120
	Pallidum	1.23 (1.19 to 1.25)	1.15 (0.99 to 1.25)	2168.5, 0.106	0.283
	Hippocampus	4.10 (4.05 to 4.15)	4.08 (3.79 to 4.51)	2823, 0.857	0.914
	Amygdala	1.48 (1.45 to 1.50)	1.47 (1.30 to 1.57)	2676.5, 0.617	0.705
	Nucleus accumbens	0.52 (0.50 to 0.53)	0.44 (0.40 to 0.49)	1668, 0.007	0.072
Right	Lateral ventricle	7.17 (6.83 to 7.64)	8.57 (7.48 to 10.58)	2257, 0.159	0.363
	Thalamus	6.28 (6.25 to 6.35)	6.11 (5.83 to 6.70)	2537, 0.420	0.611
	Caudate	3.30 (3.26 to 3.36)	3.21 (3.04 to 3.44)	2462, 0.338	0.541
	Putamen	4.64 (4.58 to 4.71)	4.31 (3.88 to 4.63)	1713, 0.009	0.072
	Pallidum	1.40 (1.39 to 1.42)	1.31 (1.16 to 1.40)	1816.5, 0.019	0.101
	Hippocampus	4.23 (4.20 to 4.27)	4.19 (3.97 to 4.60)	2903, 0.997	0.997
	Amygdala	1.53 (1.51 to 1.55)	1.46 (1.37 to 1.76)	2635, 0.554	0.682
	Nucleus accumbens	0.54 (0.53 to 0.55)	0.50 (0.45 to 0.60)	2460, 0.329	0.541

Data are presented as median values with 95% confidence limits.

## Discussion

We aimed to refine and extend the list of medical and neurobehavioural phenotypes linked to XLI-associated genetic mutations using an approach arguably less prone to confounding than those undertaken previously. The prevalence of the mutation-of-interest in our sample was ~1/730 females, a figure consistent with published estimates of 1/750–1200 based on prenatal screening data and mutation transmission rates.^6 31^ The prevalence of the mutation among males in our sample was ~1/2200, a figure slightly lower than the ~1/1500 estimate arising from prenatal screening in North American populations.^32 33^ Although the lower prevalence in our sample could reflect geographical genetic differences, it more likely reflects an ascertainment bias whereby some males with XLI-associated genetic deletions are not recruited into the UK Biobank; this could be due, in part, to psychological characteristics of this group.^34^ Our experimental strategy meant that individuals with small deletions or point mutations within *STS* would have been included in the non-deletion carrier ‘control’ group; however, the inclusion of these rare individuals would have negligible effects on the overall group score, and would likely act to reduce the magnitude of between-group effects.

Unsurprisingly, the prevalence of relevant skin phenotypes based on ICD-10 and self-report diagnostic data was significantly higher in male deletion carriers relative to non-carriers. However,<6% of male deletion carriers reported a relevant diagnosis, implying that older STS-deficient individuals are only rarely being diagnosed with XLI. This may be because the dermatological manifestations of the condition are frequently mild with only more severe and/or visible cases recognised, and some cases could have been misdiagnosed as eczema/dermatitis.^35^ Within male deletion subjects, we found no evidence for increased rates of phenotypes most closely related to those previously suggested to be elevated in individuals with XLI (testicular maldescent and cataracts); given reliable existing data on an association between XLI and cryptorchidism in paediatric patients,^1^ the lack of association with testicular maldescent observed currently may be explained by an unwillingness or inability of older participants to self-report this condition.

We identified one robust novel medical phenotype, atrial fibrillation/flutter, presenting more commonly in male deletion carriers (10.5%) than in controls (2.7%); importantly, the prevalence rate of atrial fibrillation/flutter in our controls is comparable with that observed in epidemiological studies across Europe and the USA (2%–3%).^36^ Possible mechanism(s) underlying the relationship between XLI-associated mutations and heart arrhythmia are manifold and might be investigated in experimentally tractable systems. It is noteworthy that XLI impacts on circulating DHEAS levels,^37 38^ which have repeatedly been linked to atrial fibrillation in older men,^39–41^ and that paroxysmal supraventricular tachycardia has been noted in a young boy with XLI.^42^ DHEAS may feasibly influence atrial fibrillation through conversion to biologically active androgens and oestrogens, via vascular remodelling, or through its anti-inflammatory action.^40^ It is also of interest that microarray analysis has implicated reduced triadin expression in STS-deficient mouse tissues^43^ as, in man, the absence of cardiac triadin is associated with increased risk of arrhythmia.^44 45^ Although prescription rates for medication used to treat heart arrhythmias did not differ across groups, this might be explained by the fact that these medications are used to treat other conditions.

We found no evidence that male or female deletion carriers differed from their respective controls in terms of rates of psychiatric/neurological diagnoses, and prescription rates for medications used to treat ADHD and mood disorders were equivalent across groups. However, these findings should be viewed in light of low levels of psychopathology within the UK Biobank sample,^18^ and in particular, the very low baseline rate of developmental disorder diagnoses. MHQ analysis revealed that both male and female deletion carrier groups were affected by higher rates of irritability than their respective controls, an observation in line with our previous findings,^6 12^ and with elevated levels of aggression in STS-deficient mice.^16 46^ Male deletion carriers were more likely than male controls to report experiencing psychological distress and a variety of depressive and anxiety-related symptoms, and mood symptoms may be under-recognised within this group. The increased rate of depressive and anxiety-related traits in male deletion carriers is unlikely to be due to increased exposure to traumatic events, but may feasibly be related to having to live with a potentially stigmatising, lifelong skin condition; however, the fact that *Sts*-deficient mice exhibit anxiety-related traits^16^ suggests that biological influences contribute. Female deletion carriers showed some evidence for differences in mood symptoms from controls, but these differences were smaller in magnitude and less consistent in pattern/direction than those observed for males, in accordance with the idea that female deletion carriers exhibit milder phenotypes than male deletion carriers.

Given data suggesting increased rates of ADHD^3 9 10^ and inattention in male^12^ and female^6^ deletion carriers, and attentional abnormalities in STS-deficient mice,^17^ we anticipated group differences on attentionally demanding cognitive tasks. Although we did find some evidence that male deletion carriers exhibited slower reaction times than male controls on a simple stimulus-response task (consistent with impaired attention, reduced motor impulsivity and/or slower processing speed), the two groups did not differ on the Trail Making Tasks. This lack of effect could be explained by low power. We did find evidence across both male and female deletion groups for poorer performance on the Fluid Intelligence Task, taxing multiple high-level cognitive skills; this performance deficit did not translate into impairments in academic performance.

We examined, for the first time, the effects of XLI-associated genetic mutations on neuroanatomy, although only in female carriers. Our analyses provide preliminary evidence for reduced right putamen and pallidum volume, and left accumbens volume, but no other subcortical structural changes, in deletion carriers relative to controls. We argue that these specific structural differences are genuine, and could partially explain effects on disorder risk/cognition/behaviour: a) *STS* is highly expressed in the basal ganglia during human neurodevelopment^8^ and adult STS-deficient male mice exhibit substantially altered striatal neurochemistry,^47^ b) reduced volume of the putamen/nucleus accumbens is observed in idiopathic autism^48^ and ADHD^49^ cases (with reduced pallidum volume also seen in the former), c) adult basal ganglia volumes correlate with aspects of intelligence^50^ and this structure mediates attention/distractibility.^51^ Future functional neuroimaging studies in male and female deletion carriers might examine the extent to which the structure, function and neurochemistry of the basal ganglia correlates with behavioural and cognitive measures. As basal ganglia serotonergic (5-HT) function mediates attention, impulsivity and mood phenotypes (notably via 5-HT_2A_ and 5-HT_2C_ receptors)^52 53^ and is altered in STS-deficient mice,^47^ a particular focus on this system may be warranted.

In summary, this study has highlighted a novel medical phenotype coupled with XLI-associated mutations, has confirmed an excess of depression and anxiety-related traits in male carriers, and has, for the first time, identified cognitive and neuroanatomical correlates of these genetic differences. Our findings indicate that individuals with XLI might benefit from multidisciplinary clinical care from dermatologists, psychiatrists, and cardiologists, and should lead to improved genetic counselling for patients and their families.
