# Supplementary material for: Medical and neurobehavioural phenotypes in carriers of X-linked ichthyosis-associated genetic deletions in the UK Biobank
Source: J Med Genet. 2020 Mar 5;57(10):692–8. doi: 10.1136/jmedgenet-2019-106676 (PMC7525778; doi:10.1136/jmedgenet-2019-106676)
Supplement: Supplementary data [file jmedgenet-2019-106676supp001.pdf]

Supplementary Table 1. ICD-10 medical diagnoses in male deletion carriers and male controls.

UK Biobank diagnosis code	ICD-10 descriptive code	Male controls affected	Male controls unaffected	Male deletion carriers affected	Male deletion carriers unaffected	Prevalence in male controls	Prevalence in male deletion carriers	Statistics	Benjamini-Hochberg corrected p-value (FDR<0.1)
1429	Skin of other and unspecified parts of face	2880	187697	5	81	1.5	5.8	p=0.01	0.045
1522	Malignant neoplasm of prostate	5192	185385	3	83	2.7	3.5	p=0.51	0.653
3704	Cataract, unspecified	5573	185004	3	83	2.9	3.5	p=0.742	0.742
4231	Atrial fibrillation and flutter	5193	185384	9	77	2.7	10.5	p=0.001	0.009
5004	Unilateral or unspecified inguinal hernia, without obstruction or gangrene	13031	177546	4	82	6.8	4.7	$\chi2[1]=0.348$, p=0.555	0.653
5058	Non-infective gastro-enteritis and colitis, unspecified	3930	186647	3	83	2.1	3.5	p=0.262	0.59
5078	Diverticular disease of large intestine without perforation or abscess	5645	184932	5	81	3.0	5.8	p=0.112	0.336
12318	Other and unspecified abdominal pain	4614	185963	3	83	2.4	3.5	p=0.467	0.653
12388	Unspecified haematuria	7459	183118	4	82	3.9	4.7	p=0.581	0.653

Supplementary Table 2. ICD-10 medical diagnoses in female deletion carriers and female controls.

UK Biobank diagnosis code	ICD-10 descriptive code	Female controls affected	Female controls unaffected	Female deletion carriers affected	Female deletion carriers unaffected	Prevalence in female controls	Prevalence in female deletion carriers	Statistics	Benjamini-Hochberg corrected p-value (FDR<0.1)
1945	Leiomyoma of uterus, unspecified	5357	222505	9	303	2.4	2.9	$\chi2[1]=0.189$, p=0.664	1.000
3401	Carpal tunnel syndrome	6662	221200	9	303	2.9	2.9	$\chi2[1]=0.000$, p>0.99	1.000
3704	Cataract, unspecified	7141	220721	13	299	3.1	4.2	$\chi2[1]=0.781$, p=0.377	1.000
4429	Varicose veins of lower extremities without ulcer or inflammation	6948	220914	8	304	3.0	2.6	$\chi2[1]=0.111$, p=0.739	1.000
5029	Diaphragmatic hernia without obstruction or gangrene	6265	221597	12	300	2.7	3.8	$\chi2[1]=1.021$, p=0.312	1.000
5058	Non-infective gastro-enteritis and colitis, unspecified	6536	221326	9	303	2.9	2.9	$\chi2[1]=0.000$, p>0.99	1.000
6570	Gonarthrosis, unspecified	5379	222483	9	303	2.4	2.9	$\chi2[1]=0.179$, p=0.673	1.000
10640	Postmenopausal bleeding	7099	220763	11	301	3.1	3.5	$\chi2[1]=0.064$, p=0.800	1.000
12318	Other and unspecified abdominal pain	8539	219323	11	301	3.7	3.5	$\chi2[1]=0.003$, p=0.955	1.000
12461	Headache	3294	224568	8	304	1.4	2.6	p=0.097	0.970
1429	Skin of other and unspecified parts of face	2804	225058	1	311	1.2	0.3	p=0.196	-
4231	Atrial fibrillation and flutter	2551	225311	3	309	1.1	1.0	p>0.99	-

Supplementary Table 3. ICD-10 mental health diagnoses in male deletion carriers and male controls.

Class of disorder	Subclass of disorder	UK Biobank diagnosis code	ICD-10 descriptive code	Male controls affected	Male controls unaffected	Male deletion carriers affected	Male deletion carriers unaffected	Prevalence in male controls	Prevalence in male deletion carriers	P-value
Developmental disorders	Autism-related disorders	3210	Childhood autism							
		3121	Atypical autism							
		3123	Other childhood disintegrative disorder	20	190557	0	86	0.0	0.0	>0.99
		3125	Asperger's syndrome							
		3126	Other pervasive developmental disorders							
		3127	Pervasive developmental disorder, unspecified							
	Hyperkinetic disorder	3131	Disturbance of activity and attention							
		3132	Hyperkinetic conduct disorder	0	190577	0	86	0.0	0.0	>0.99
		3133	Other hyperkinetic disorders							
		3134	Hyperkinetic disorder, unspecified							
	Conduct disorders	3136	Conduct disorder confined to the family context							
		3137	Unsocialised conduct disorder							
		3138	Socialised conduct disorder							
		3139	Oppositional defiant disorder							
		3140	Other conduct disorders	2	190575	0	86	0.0	0.0	>0.99
		3141	Conduct disorder, unspecified							
		3143	Depressive conduct disorder							
		3144	Other mixed disorders of conduct and emotions							
		3145	Mixed disorder of conduct and emotions, unspecified							
	Dyslexia and alexia	12446	Dyslexia and alexia	0	190577	0	86	0.0	0.0	>0.99
	Schizophrenia	2864	Paranoid schizophrenia							
		2865	Hebephrenic schizophrenia							
		2866	Catatonic schizophrenia							
		2867	Undifferentiated schizophrenia							
		2868	Postschizophrenic depression	175	190402	0	86	0.1	0.0	>0.99
		2869	Residual schizophrenia							
		2870	Simple schizophrenia							
		2871	Other schizophrenia							
		2872	Schizophrenia, unspecified							
		2873	Schizotypal disorder							

**Mood and anxiety disorders**	Mania/bipolar disorder	2895	Hypomania							
		2896	Mania without psychotic symptoms							
		2897	Mania with psychotic symptoms							
		2898	Other manic episodes							
		2899	Manic episode, unspecified							
		2901	Bipolar affective disorder, current episode hypomanic							
		2902	Bipolar affective disorder, current episode manic without psychotic symptoms							
		2903	Bipolar affective disorder, current episode manic with psychotic symptoms	214	190363	0	86	0.1	0.0	>0.99
		2904	Bipolar affective disorder, current episode mild or moderate depression							
		2905	Bipolar affective disorder, current episode severe depression without psychotic symptoms							
		2906	Bipolar affective disorder, current episode severe depression with psychotic symptoms							
		2907	Bipolar affective disorder, current episode mixed							
		2908	Bipolar affective disorder, currently in remission							
		2909	Other bipolar affective disorders							
		2910	Bipolar affective disorder, unspecified							
	Depressive disorder	2912	Mild depressive episode							
		2913	Moderate depressive episode							
		2914	Severe depressive episode without psychotic symptoms							
		2915	Severe depressive episode with psychotic symptoms							
		2916	Other depressive episodes							
		2917	Depressive episode, unspecified							
		2919	Recurrent depressive disorder, current episode mild	587	189990	0	86	0.3	0.0	>0.99
		2920	Recurrent depressive disorder, current episode moderate							
		2921	Recurrent depressive disorder, current episode severe without psychotic symptoms							
		2922	Recurrent depressive disorder, current episode severe with psychotic symptoms							
		2923	Recurrent depressive disorder, currently in remission							
		2924	Other recurrent depressive disorders							
		2925	Recurrent depressive disorder, unspecified							
		2937	Agoraphobia							

Anxiety disorder									
	2938	Social phobias							
	2939	Specific (isolated) phobias							
	2940	Other phobic anxiety disorders							
	2941	Phobic anxiety disorder, unspecified							
	2943	Panic disorder [episodic paroxysmal anxiety]	236	190341	0	86	0.1	0.0	>0.99
	2944	Generalised anxiety disorder							
	2945	Mixed anxiety and depressive disorder							
	2946	Other mixed anxiety disorders							
	2947	Other specified anxiety disorders							
	2948	Anxiety disorder, unspecified							
Obsessive Compulsive Disorder	2950	Predominantly obsessional thoughts or ruminations							
	2951	Predominantly compulsive acts [obsessional rituals]							
	2952	Mixed obsessional thoughts and acts	19	190558	0	86	0.0	0.0	>0.99
	2953	Other obsessive-compulsive disorders							
	2954	Obsessive-compulsive disorder, unspecified							

Supplementary Table 4. ICD-10 mental health diagnoses in female deletion carriers and female controls.

		UK Biobank diagnosis code	ICD-10 descriptive code	Female controls affected	Female controls unaffected	Female deletion carriers affected	Female deletion carriers unaffected	Prevalence in female controls	Prevalence in female deletion carriers	P-value
**Developmental disorders**	Autism-related disorders	3210	Childhood autism							
		3121	Atypical autism							
		3123	Other childhood disintegrative disorder	20	227842	0	312	0.0	0.0	>0.99
		3125	Asperger's syndrome							
		3126	Other pervasive developmental disorders							
		3127	Pervasive developmental disorder, unspecified							
	Hyperkinetic disorder	3131	Disturbance of activity and attention							
		3132	Hyperkinetic conduct disorder	0	227862	0	312	0.0	0.0	>0.99
		3133	Other hyperkinetic disorders							
		3134	Hyperkinetic disorder, unspecified							
	Conduct disorders	3136	Conduct disorder confined to the family context							
		3137	Unsocialised conduct disorder							
		3138	Socialised conduct disorder							
		3139	Oppositional defiant disorder							
		3140	Other conduct disorders	0	227862	0	312	0.0	0.0	>0.99
		3141	Conduct disorder, unspecified							
		3143	Depressive conduct disorder							
		3144	Other mixed disorders of conduct and emotions							
		3145	Mixed disorder of conduct and emotions, unspecified							
	Dyslexia and alexia	12446	Dyslexia and alexia	2	227860	0	312	0.0	0.0	>0.99
	Schizophrenia	2864	Paranoid schizophrenia							
		2865	Hebephrenic schizophrenia							
		2866	Catatonic schizophrenia							
		2867	Undifferentiated schizophrenia							
		2868	Postschizophrenic depression	87	227775	0	312	0.0	0.0	>0.99
		2869	Residual schizophrenia							
		2870	Simple schizophrenia							
		2871	Other schizophrenia							
		2872	Schizophrenia, unspecified							
		2873	Schizotypal disorder							

**Mood and anxiety disorders**	Mania/bipolar disorder	2895	Hypomania							
		2896	Mania without psychotic symptoms							
		2897	Mania with psychotic symptoms							
		2898	Other manic episodes							
		2899	Manic episode, unspecified							
		2901	Bipolar affective disorder, current episode hypomanic							
		2902	Bipolar affective disorder, current episode manic without psychotic symptoms							
		2903	Bipolar affective disorder, current episode manic with psychotic symptoms	346	227516	0	312	0.2	0.0	>0.99
		2904	Bipolar affective disorder, current episode mild or moderate depression							
		2905	Bipolar affective disorder, current episode severe depression without psychotic symptoms							
		2906	Bipolar affective disorder, current episode severe depression with psychotic symptoms							
		2907	Bipolar affective disorder, current episode mixed							
		2908	Bipolar affective disorder, currently in remission							
		2909	Other bipolar affective disorders							
		2910	Bipolar affective disorder, unspecified							
	Depressive disorder	2912	Mild depressive episode							
		2913	Moderate depressive episode							
		2914	Severe depressive episode without psychotic symptoms							
		2915	Severe depressive episode with psychotic symptoms							
		2916	Other depressive episodes							
		2917	Depressive episode, unspecified							
		2919	Recurrent depressive disorder, current episode mild	759	227103	1	311	0.3	0.3	>0.99
		2920	Recurrent depressive disorder, current episode moderate							
		2921	Recurrent depressive disorder, current episode severe without psychotic symptoms							
		2922	Recurrent depressive disorder, current episode severe with psychotic symptoms							
		2923	Recurrent depressive disorder, currently in remission							
		2924	Other recurrent depressive disorders							
		2925	Recurrent depressive disorder, unspecified							

Anxiety disorder	2937	Agoraphobia							
	2938	Social phobias							
	2939	Specific (isolated) phobias							
	2940	Other phobic anxiety disorders							
	2941	Phobic anxiety disorder, unspecified							
	2943	Panic disorder [episodic paroxysmal anxiety]	365	227497	1	311	0.2	0.3	0.394
	2944	Generalised anxiety disorder							
	2945	Mixed anxiety and depressive disorder							
	2946	Other mixed anxiety disorders							
	2947	Other specified anxiety disorders							
	2948	Anxiety disorder, unspecified							
Obsessive Compulsive Disorder	2950	Predominantly obsessional thoughts or ruminations							
	2951	Predominantly compulsive acts [obsessional rituals]							
	2952	Mixed obsessional thoughts and acts	14	227848	0	312	0.0	0.0	>0.99
	2953	Other obsessive-compulsive disorders							
	2954	Obsessive-compulsive disorder, unspecified							

Supplementary Table 5. Non-cancer illnesses in male deletion carriers and male controls.

Body system	UK Biobank non-cancer illness code	Non-cancer illness	Male controls affected	Male controls unaffected	Male deletion carriers affected	Male deletion carriers unaffected	Prevalence in male controls	Prevalence in male deletion carriers	P-value	Benjamini-Hochberg corrected p-value (FDR<0.1)
Heart and cardiovascular	1066	Heart/cardiac problem	688	189889	0	86	0.4	0.0	>0.99	>0.99
	1077	Heart arrythmia	1167	189410	3	83	0.6	3.5	0.016	0.208
	1471	Atrial fibrillation	2355	188222	1	85	1.2	1.2	>0.99	>0.99
	1483	Atrial flutter	72	190505	1	85	0.0	1.2	0.032	0.250
	1485	Irregular heartbeat	412	190165	0	86	0.2	0.0	>0.99	>0.99
Reproductive	1214	Testicular problems (not cancer)	593	189984	0	86	0.3	0.0	>0.99	>0.99
	1404	Male infertility	27	190550	0	86	0.0	0.0	>0.99	>0.99
	1679	Undescended testicle	188	190389	0	86	0.1	0.0	>0.99	>0.99
Thyroid	1224	Thyroid problem (not cancer)	164	190413	0	86	0.1	0.0	>0.99	>0.99
	1225	Hyperthyroidism	586	189991	0	86	0.3	0.0	>0.99	>0.99
	1226	Hypothyroidism	3085	187492	1	85	1.6	1.2	>0.99	>0.99
Eye	1278	Cataract	2993	187584	3	83	1.6	3.5	0.154	0.858
Immune system	1374	Allergy/hypersensitivity/anaphylaxis	1015	189562	4	82	0.5	4.7	0.001	0.020
Skin	1452	Eczema/dermatitis	5108	185469	6	80	2.7	7.0	0.028	0.250
	1453	Psoriasis	2628	187949	1	85	1.4	1.2	>0.99	>0.99
	1454	Blistering/desquamating skin disorder	325	190252	4	82	0.2	4.7	<0.001	<0.020
Nervous										

system	1243	Psychological/psychiatric problem	167	190410	0	86	0.1	0.0	>0.99	>0.99
	1258	Chronic/degenerative neurological problem	66	190511	0	86	0.0	0.0	>0.99	>0.99
	1259	Motor neurone disease	34	190543	0	86	0.0	0.0	>0.99	>0.99
	1261	Multiple sclerosis	387	190190	0	86	0.2	0.0	>0.99	>0.99
	1262	Parkinson's Disease	485	190092	0	86	0.3	0.0	>0.99	>0.99
	1263	Dementia/Alzheimer's/cognitive impairment	65	190512	0	86	0.0	0.0	>0.99	>0.99
	1264	Epilepsy	1709	188868	1	85	0.9	1.2	0.539	>0.99
	1265	Migraine	2894	187683	2	84	1.5	2.3	0.376	>0.99
	1286	Depression	8541	182036	2	84	4.5	2.3	0.595	>0.99
	1287	Anxiety/panic attacks	2120	188457	3	83	1.1	3.5	0.072	0.468
	1288	Nervous breakdown	275	190302	0	86	0.1	0.0	>0.99	>0.99
	1289	Schizophrenia	304	190273	0	86	0.2	0.0	>0.99	>0.99
	1290	Deliberate self-harm/suicide	82	190495	0	86	0.0	0.0	>0.99	>0.99
	1291	Mania/bipolar disorder	508	190069	0	86	0.3	0.0	>0.99	>0.99
	1408	Alcohol dependency	460	190117	0	86	0.2	0.0	>0.99	>0.99
	1409	Opioid dependency	20	190557	0	86	0.0	0.0	>0.99	>0.99
	1410	Other substance abuse	34	190543	0	86	0.0	0.0	>0.99	>0.99
	1469	Posttraumatic Stress Disorder	145	190432	0	86	0.1	0.0	>0.99	>0.99
	1470	Anorexia/bulimia	15	190562	0	86	0.0	0.0	>0.99	>0.99
	1531	Postnatal depression	1	190576	0	86	0.0	0.0	>0.99	>0.99
	1614	Stress	267	190310	0	86	0.1	0.0	>0.99	>0.99
	1615	Obsessive Compulsive Disorder	49	190528	0	86	0.0	0.0	>0.99	>0.99
	1616	Insomnia	119	190458	0	86	0.1	0.0	>0.99	>0.99

Supplementary Table 6. Non-cancer illnesses in female deletion carriers and female controls.

Body system	UK Biobank non-cancer illness code	Non-cancer illness	Female controls affected	Female controls unaffected	Female deletion carriers affected	Female deletion carriers unaffected	Prevalence in female controls	Prevalence in female deletion carriers	P-value	Benjamini-Hochberg corrected p-value (FDR<0.1)
Heart and cardiovascular	1066	Heart/cardiac problem	666	227196	2	310	0.3	0.6	0.232	>0.99
	1077	Heart arrythmia	1147	226715	2	310	0.5	0.6	0.673	>0.99
	1471	Atrial fibrillation	1084	226778	0	312	0.5	0.0	0.412	>0.99
	1483	Atrial flutter	26	227836	0	312	0.0	0.0	>0.99	>0.99
	1485	Irregular heartbeat	389	227473	0	312	0.2	0.0	>0.99	>0.99
Reproductive	1402	Endometriosis	3556	224306	1	311	1.6	0.3	0.102	>0.99
	1403	Female infertility	516	227346	1	311	0.2	0.3	0.507	>0.99
Thyroid	1224	Thyroid problem (not cancer)	946	226916	1	311	0.4	0.3	>0.99	>0.99
	1225	Hyperthyroidism	2695	225167	5	307	1.2	1.6	0.426	>0.99
	1226	Hypothyroidism	17905	209957	23	289	7.9	7.4	$\chi 2[1]=0.046$, p=0.831	>0.99
Eye	1278	Cataract	3921	223941	6	306	1.7	1.9	$\chi 2[1]=0.003$, p=0.955	>0.99
Immune system	1374	Allergy/hypersensitivity/anaphylaxis	2035	225827	1	311	0.9	0.3	0.536	>0.99
Hair	1667	Alopecia/hair loss	66	227796	0	312	0.0	0.0	>0.99	>0.99
Skin	1452	Eczema/dermatitis	6257	221605	9	303	2.7	2.9	$\chi 2[1]=0.000$, p>0.99	>0.99
	1453	Psoriasis	2323	225539	6	306	1.0	1.9	0.143	>0.99
	1454	Blistering/desquamating skin disorder	336	227526	0	312	0.1	0.0	>0.99	>0.99
Nervous system	1243	Psychological/psychiatric problem	219	227643	0	312	0.1	0.0	>0.99	>0.99
	1258	Chronic/degenerative neurological problem	81	227781	0	312	0.0	0.0	>0.99	>0.99

1259	Motor neurone disease	14	227848	0	312	0.0	0.0	>0.99	>0.99
1261	Multiple sclerosis	1135	226727	3	309	0.5	1.0	0.205	>0.99
1262	Parkinson's Disease	283	227579	1	311	0.1	0.3	0.322	>0.99
1263	Dementia/Alzheimer's/cognitive impairment	51	227811	0	312	0.0	0.0	>0.99	>0.99
1264	Epilepsy	1775	226087	3	309	0.8	1.0	0.526	>0.99
1265	Migraine	10083	217779	7	305	4.4	2.2	$\chi2[1]=3.011$, p=0.083	>0.99
1286	Depression	16496	211366	27	285	7.2	8.7	$\chi2[1]=0.729$, p=0.393	>0.99
1287	Anxiety/panic attacks	3959	223903	6	306	1.7	1.9	$\chi2[1]=0.001$, p=0.973	>0.99
1288	Nervous breakdown	366	227496	0	312	0.2	0.0	>0.99	>0.99
1289	Schizophrenia	147	227715	0	312	0.1	0.0	>0.99	>0.99
1290	Deliberate self-harm/suicide	110	227752	0	312	0.0	0.0	>0.99	>0.99
1291	Mania/bipolar disorder	657	227205	1	311	0.3	0.3	0.594	>0.99
1408	Alcohol dependency	177	227685	0	312	0.1	0.0	>0.99	>0.99
1409	Opioid dependency	5	227857	0	312	0.0	0.0	>0.99	>0.99
1410	Other substance abuse	14	227848	0	312	0.0	0.0	>0.99	>0.99
1469	Posttraumatic Stress Disorder	150	227712	0	312	0.1	0.0	>0.99	>0.99
1470	Anorexia/bulimia	314	227548	0	312	0.1	0.0	>0.99	>0.99
1531	Postnatal depression	401	227461	0	312	0.2	0.0	>0.99	>0.99
1614	Stress	374	227488	2	310	0.2	0.6	0.094	>0.99
1615	Obsessive Compulsive Disorder	48	227814	0	312	0.0	0.0	>0.99	>0.99
1616	Insomnia	296	227566	0	312	0.1	0.0	>0.99	>0.99

Supplementary Table 7. Mental Health Questionnaire (MHQ) responses in male deletion carriers and male controls.

Depression

Dual answer questions	**Male control**	**Male deletion**	**Statistics**
Ever had prolonged feelings of sadness or depression? (Yes/No)	25669(44%)/33052(56%)	15(75%)/5(25%)	χ2[1]=6.733, p=0.009
Ever had prolonged loss of interest in normal activities? (Yes/No)	18460(31%)/40257(69%)	13(62%)/8(38%)	χ2[1]=7.680, p=0.006
Depression possibly related to stressful or traumatic event (Yes/No)	17354/9770	10/6	χ2[1]=0.000, p>0.99
Feelings of tiredness during worst episode of depression (Yes/No)	18384/5915	12/3	p>0.99
Did your sleep change? (Yes/No)	17353/6075	12/4	p>0.99
Trouble falling asleep (Yes/No)	13225/4128	8/4	p=0.496
Sleeping too much (Yes/No)	3417/13936	3/9	p=0.714
Waking too early (Yes/No)	13086/4267	8/4	p=0.505
Difficulty concentrating during worst depression (Yes/No)	18463/6009	15/1	p=0.141
Feelings of worthlessness during worst episode of depression (Yes/No)	12321/13251	11/5	χ2[1]=1.948, p=0.163
Thoughts of death during worst depression (Yes/No)	11891/13902	5/10	χ2[1]=0.537, p=0.464
Depression possibly related to childbirth (Yes/No)	-	-	-
Professional informed about depression (Yes/No)	15373/11745	8/8	χ2[1]=0.083, p=0.774
Substances taken for depression (unprescribed)(Yes/No)	1236/57619	1/20	p=0.360
Substances taken for depression (prescribed)(Yes/No)	9914/48941	5/16	p=0.382
Substances taken for depression (drugs or alcohol)(Yes/No)	4566/54289	2/19	p=0.676
Talking therapies (Yes/No)	9046/49809	5/16	p=0.356
Other non-drug therapies (e.g. yoga) (Yes/No)	2334/56521	2/19	p=0.202
Multiple answer questions			
Trouble falling or staying asleep, or sleeping too much (1: not at all-4:nearly every day)	1.74±0.006	2.07±0.267	U=519025.5, p=0.153
Recent feelings of inadequacy (1: not at all-4:nearly every day)	1.34±0.005	1.57±0.228	U=511192, p=0.039
Recent trouble concentrating on things (1: not at all-4:nearly every day)	1.33±0.005	1.57±0.228	U=487101, p=0.011
Recent feelings of depression (1: not at all-4:nearly every day)	1.36±0.005	1.64±0.248	U=519805.5, p=0.072
Recent poor appetite or overeating (1: not at all-4:nearly every day)	1.28±0.005	1.36±0.225	U=582574.5, p=0.450
Recent thoughts of suicide or self-harm (1: not at all-4:nearly every day)	1.10±0.003	1.29±0.221	U=552023.5, p=0.024
Recent lack of interest or pleasure in doing things (1: not at all-4:nearly every day)	1.34±0.005	1.57±0.228	U=490482.5, p=0.015
Recent changes in speed/amount of moving or speaking (1: not at all-4:nearly every day)	1.11±0.003	1.36±0.225	U=561630, p=0.069
Recent feelings of tiredness or low energy (1: not at all-4:nearly every day)	1.76±0.006	2.00±0.234	U=499865.5, p=0.196
Fraction of day affected during worst episode of depression (1:less than half the day-4:all day long)	2.76±0.007	2.93±0.245	U=177823.5, p=0.614
Frequency of depressed days during worst episode of depression (1:less often-3:every day)	2.23±0.004	2.21±0.214	U=180055.5, p=0.510

Weight change during worst episode of depression (0: stayed about the same or was on a diet, 1:gained weight, 2:lost weight, 3:both gained and lost weight)	0:12603, 1:3055, 2:5651, 3:1175	0:13, 1:2, 2:1, 3:0	p=0.213
Duration of worst depression (1:less than a month-6:over two years)	2.88±0.011	3.07±0.438	U=201796, p=0.660
Impact on normal roles during worst episode of depression (0:not at all-3:a lot)	1.95±0.006	2.00±0.234	U=215475.5, p=0.949
Age at first episode of depression (yrs)	39.29±0.104	37.71±4.876	U=185977.5, p=0.659
Age at last episode of depression (yrs)	49.30±0.091	55.21±2.689	U=145534, p=0.188
Lifetime number of depressed periods	145.17±2.420	287.79±124.757	U=147693.5, p=0.072
**Mania**			
Dual answer questions			
Manifestations of mania (more talkative than usual) (Yes/No)	2896/55959	2/19	p=0.277
Manifestations of mania (more restless than usual)(Yes/No)	6607/52248	5/16	p=0.079
Manifestations of mania (my thoughts were racing)(Yes/No)	5337/53518	4/17	p=0.117
Manifestations of mania (needed less sleep than usual)(Yes/No)	1978/56877	2/19	p=0.156
Manifestations of mania (more creative than usual)(Yes/No)	1902/56953	0/21	p>0.99
Manifestations of mania (was easily distracted)(Yes/No)	4551/54304	4/17	p=0.074
Manifestations of mania (was more confident than usual)(Yes/No)	1992/56863	0/21	p>0.99
Manifestations of mania (was more active than usual)(Yes/No)	3019/55836	3/18	p=0.090
Ever had a period of mania/excitability? (Yes/No)	2814/54385	1/20	p>0.99
Ever had a period of extreme irritability? (Yes/No)	14245(25%)/43013(75%)	10(48%)/11(52%)	$\chi 2[1]=4.654$, p=0.031
Severity of problems due to mania or irritability (No problems/caused problems)	8987/4173	4/5	p=0.153
Multiple answer questions			
Longest period of mania or irritability (1: less than 24hrs-3: a week or more)	1.86±0.007	2.13±0.350	U=45749, p=0.396
**Anxiety**			
Dual answer questions			
Ever felt worried, tense or anxious for most of a month or longer (Yes/No)	11828(21%)/44247(79%)	9(43%)/12(57%)	p=0.027
Ever worried more than most people would in similar situation (Yes/No)	10750/39424	7/10	p=0.069
Stronger worrying (than other people) during period of worst anxiety (Yes/No)	8585/1581	6/1	p>0.99
Worried most days during period of worst anxiety (Yes/No)	12058/2097	8/1	p>0.99

Number of things worried about during worst period of anxiety (One thing/More than one thing)	6743/7534	2/6	p=0.295
Difficulty stopping worrying during worst period of anxiety (Yes/No)	13174/1095	7/1	p=0.472
Multiple worries during worst period of anxiety (Yes/No)	10070/4067	7/2	p>0.99
Tense, sore or aching muscles during worse period of anxiety (Yes/No)	4173(31%)/9314(69%)	6(75%)/2(25%)	p=0.013
Difficulty concentrating during worst period of anxiety (Yes/No)	10560/3599	9/0	p=0.124
More irritable than usual during worst period of anxiety (Yes/No)	9967/3686	8/1	p=0.460
Restless during period of worst anxiety (Yes/No)	8577/5227	4/4	p=0.487
Keyed up or on edge during worst period of anxiety (Yes/No)	10917/3139	8/1	p=0.694
Frequent trouble falling or staying asleep during worst period of anxiety (Yes/No)	10850(76%)/3464(24%)	4(44%)/5(56%)	p=0.043
Easily tired during worst period of anxiety (Yes/No)	8955/4786	6/3	p>0.99
Professional informed about anxiety (Yes/No)	8376/6254	5/4	p>0.99
Substances taken for anxiety (unprescribed)(Yes/No)	901/57954	1/20	p=0.277
Substances taken for anxiety (prescribed)(Yes/No)	5748/53107	4/17	p=0.143
Substances taken for anxiety (drugs or alcohol) (Yes/No)	3191/55664	2/19	p=0.317
Activities undertaken to treat anxiety (talking therapies)(Yes/No)	5495/53360	3/18	p=0.440
Activities undertaken to treat anxiety (other therapeutic activities e.g. yoga)(Yes/No)	1657/57198	1/20	p=0.451
Multiple answer questions			
Recent easy annoyance or irritability (1: not at all-4:nearly every day)	1.31±0.002	1.86±0.210	U=405199, p<0.001
Recent feelings of nervousness or anxiety (1: not at all-4:nearly every day)	1.28±0.002	1.62±0.234	U=536245, p=0.159
Recent inability to stop or control worrying (1: not at all-4:nearly every day)	1.23±0.002	1.48±0.178	U=517872.5, p=0.058
Recent feelings of foreboding (1: not at all-4:nearly every day)	1.16±0.002	1.33±0.174	U=571941, p=0.330
Recent trouble relaxing (1: not at all-4:nearly every day)	1.31±0.003	1.52±0.178	U=524358.5, p=0.109
Recent restlessness (1: not at all-4:nearly every day)	1.14±0.002	1.38±0.161	U=506824.5, p=0.008
Recent worrying too much about different things (1: not at all-4:nearly every day)	1.31±0.003	1.62±0.212	U=522739, p=0.112
Longest period spent worried or anxious (months)	158±3	563±172	U=26962.5, p=0.010
Frequency of inability to stop worrying during worst period of anxiety (0:Never-3:Often)	2.19±0.006	2.56±0.176	U=49519.5, p=0.165
Frequency of difficulty controlling worry during worst period of anxiety (0:Never-3:Often)	2.19±0.006	2.56±0.176	U=55332.5, p=0.380
Impact on normal roles during worst period of anxiety (0: Not at all-3: A lot)	1.89±0.008	2.22±0.222	U=54027.5, p=0.319

Addictions

Ever addicted to any substance or behaviour (Yes/No)	4080/54067	1/19	p>0.99
Ever addicted to alcohol (Yes/No)	1755/2025	1/0	p=0.464
Ongoing addiction to alcohol (Yes/No)	756/974	1/0	p=0.437
Ever physically dependent on alcohol (Yes/No)	551/1132	0/1	p>0.99
Ever addicted to prescription or over-the-counter medication (Yes/No)	466/3566	0/1	p>0.99
Ever addicted to illicit or recreational drugs (Yes/No)	358/3662	0/1	p>0.99
Ever addicted to a behaviour or miscellaneous	1176/2833	1/0	p=0.294
Ongoing behavioural or miscellaneous addiction (Yes/No)	524/630	1/0	p=0.455
**Alcohol use**			
Frequency of drinking alcohol (0: Never-4: 4 or more times a week)	2.87±0.005	2.90±0.194	U=588100.5, p=0.697
Amount of alcohol drunk on a typical drinking day (1: 1-2 units-5: 10 or more units)	2.11±0.005	1.52±0.190	U=407457, p=0.013
Frequency of consuming 6 or more units of alcohol (1: Never-5: daily or almost daily)	2.29±0.005	2.24±0.275	U=574833.5, p=0.931
Frequency of inability to cease drinking in last year (1: Never-5: daily or almost daily)	1.18±0.003	1.00±0.000	U=251328, p=0.203
Frequency of failure to fulfil normal expectations due to drinking alcohol in last year (1: Never-5: daily or almost daily)	1.09±0.002	1.14±0.097	U=262675, p=0.364
Frequency of needing morning drink of alcohol after heavy drinking session in last year (1: Never-5: daily or almost daily)	1.01±0.001	1.00±0.000	U=279118, p=0.759
Frequency of feeling guilt or remorse after drinking alcohol in last year (1: Never-5: daily or almost daily)	1.25±0.003	1.07±0.071	U=251369, p=0.303
Frequency of memory loss due to drinking alcohol in the last year (1: Never-5: daily or almost daily)	1.18±0.003	1.07±0.071	U=260994.5, p=0.449
Ever been injured or injured someone else through drinking alcohol (0: No, 1:Yes, but not in last year, 2:Yes, during the last year)	0:55282, 1:3206, 2:321	0:20, 1:1, 2:0	p>0.99
Ever had known person concerned about, or recommend reduction of, alcohol consumption (0: No, 1:Yes, but not in last year, 2:Yes, during the last year)	0:50820, 1:4072, 2:3847	0:19, 1:1, 2:1	p>0.99
Age when known person last commented about drinking habits (yrs)	51.3±0.2	67.0±0.0	U=464, p=0.196
**Cannabis use**			
Ever taken cannabis (0:No, 1:1-2 times, 2:3-10 times, 3:11-100 times, 4: more than 100 times)	0.52±0.004	0.14±0.101	U=517372.5, p=0.092
Maximum frequency of taking cannabis (1:Less than once a month, 2:once a month or more but not every week, 3:once a week or more, but not every day, 4:Every day)	1.70±0.008	1.00±0.000	U=9036, p=0.293
Age when last took cannabis (yrs)	33.2±0.1	23.0±2.0	U=8252, p=0.279
**Unusual and psychotic experiences**			

Dual answer questions			
Ever seen an unreal vision (Yes/No)	1562/56684	1/20	p=0.435
Ever heard an unreal voice (Yes/No)	914/57657	0/21	p>0.99
Ever believed in unreal communications or signs (Yes/No)	406/58263	0/21	p>0.99
Ever believed in unreal conspiracy against self (Yes/No)	539/58134	0/21	p>0.99
Ever talked to a health professional about unusual or psychotic experiences (Yes/No)	603/1965	0/1	p>0.99
Ever prescribed medication for unusual or psychotic experiences (Yes/No)	332/2232	0/1	p>0.99
Multiple answer questions			
Number of times seen an unreal vision	33.1±1.9	192.0±0.0	U=103, p=0.134
Number of times heard an unreal voice	69.5±3.9	-	-
Number of times believed in unreal communications or signs	75.1±5.4	-	-
Number of times believed in unreal conspiracy against self	29.7±1.8	-	-
Frequency of unusual or psychotic experiences in last year (0:Not at all-4: Nearly every day or daily)	0.54±0.020	0.00±0.000	U=896.5, p=0.516
Age when first had unusual or psychotic experience (yrs)	35.9±0.5	15.0±0.0	U=341.5, p=0.236
Distress caused by unusual or psychotic experiences (0:Not distressing at all-4: Very distressing)	1.47±0.026	0.00±0.000	U=328, p=0.186
**Traumatic events**			
Felt loved as a child (0:Never true-4: Very often true)	3.23±0.004	3.05±0.288	U=603250.5, p=0.863
Someone to take to doctor when needed as a child (0:Never true-4: Very often true)	3.76±0.003	3.90±0.066	U=479201.5, 0.459
Been in a confiding relationship as an adult (0:Never true-4: Very often true)	3.00±0.005	3.19±0.245	U=570132, p=0.613
Able to pay rent/mortgage as an adult (0:Never true-4: Very often true)	3.74±0.003	3.86±0.104	U=590821, p=0.676
Been in serious accident believed to be life-threatening (0:Never, 1:Yes, but not in last 12 months, 2:Yes, within the last 12 months)	0:50989, 1:7578, 2:209	0:20, 1:1, 2:0	p=0.545
Been involved in combat or exposed to war zone (0:Never, 1:Yes, but not in last 12 months, 2:Yes, within the last 12 months)	0:55263, 1:3429, 2:63	0:20, 1:1, 2:0	p>0.99
Diagnosed with life-threatening illness (0:Never, 1:Yes, but not in last 12 months, 2:Yes, within the last 12 months)	0:48114, 1:9006, 2:1547	0:15, 1:6, 2:0	p=0.215
Victim of physically violent crime (0:Never, 1:Yes, but not in last 12 months, 2:Yes, within the last 12 months)	0:44349, 1:14083, 2:335	0:15, 1:6, 2:0	p=0.657
Witnessed sudden violent death (0:Never, 1:Yes, but not in last 12 months, 2:Yes, within the last 12 months)	0:47486, 1:10851, 2:417	0:19, 1:2, 2:0	p=0.487
Avoided activities or situations because of previous stressful experience in past month (0:Not at all-4:Extremely)	0.23±0.003	0.38±0.201	U=571980.5, p=0.367
Repeated disturbing thoughts of stressful experience in past month (0:Not at all-4:Extremely)	0.31±0.003	0.62±0.234	U=541898.5, p=0.186
Felt very upset when reminded of stressful experience in past month (0:Not at all-4:Extremely)	0.38±0.003	0.71±0.260	U=544289, p=0.235
Felt irritable or had angry outbursts in past month (0:Not at all-4:Extremely)	0.57±0.005	1.20±0.389	U=78051, p=0.078

Felt distant from other people in past month (0:Not at all-4:Extremely)	0.64±0.006	0.30±0.153	U=91915, p=0.328
**Happiness and subjective wellbeing**			
General happiness (1:Extremely happy-6:Extremely unhappy)	2.40±0.003	2.62±0.212	U=545238, p=0.326
General happiness with own health (1:Extremely happy-6:Extremely unhappy)	2.63±0.004	2.81±0.281	U=566695, p=0.491
Belief that own life is meaningful (1:Not at all-5:an extreme amount)	3.69±0.003	3.48±0.245	U=556178.5, p=0.518

Supplementary Table 8. Mental Health Questionnaire (MHQ) responses in female deletion carriers and female controls.

Depression

Dual answer questions	**Female control**	**Female deletion**	**Statistics**
Ever had prolonged feelings of sadness or depression? (Yes/No)	48061/28148	61/33	χ2[1]=0.068, p=0.795
Ever had prolonged loss of interest in normal activities? (Yes/No)	34628/41541	46/48	χ2[1]=0.328, p=0.567
Depression possibly related to stressful or traumatic event (Yes/No)	38341/10536	46/15	χ2[1]=0.176, p=0.674
Feelings of tiredness during worst episode of depression (Yes/No)	37680/6401	53/8	χ2[1]=0.017, p=0.897
Did your sleep change? (Yes/No)	34919/7056	47/8	χ2[1]=0.0072, p=0.788
Trouble falling asleep (Yes/No)	26474/8445	33/14	χ2[1]=0.527, p=0.468
Sleeping too much (Yes/No)	7305/27614	15/32	χ2[1]=2.796, p=0.094
Waking too early (Yes/No)	26601(76%)/8318(24%)	29(62%)/18(38%)	χ2[1]=4.650, p=0.031
Difficulty concentrating during worst depression (Yes/No)	34573/8215	47/11	χ2[1]=0.000, p>0.99
Feelings of worthlessness during worst episode of depression (Yes/No)	23734/21821	32/29	χ2[1]=0.000, p>0.99
Thoughts of death during worst depression (Yes/No)	25425/20559	29/30	χ2[1]=0.667, p=0.414
Depression possibly related to childbirth (Yes/No)	5592/38405	4/50	χ2[1]=0.931, p=0.335
Professional informed about depression (Yes/No)	33629/15198	36/24	χ2[1]=1.806, p=0.179
Substances taken for depression (unprescribed)(Yes/No)	3433/73006	4/91	p>0.99
Substances taken for depression (prescribed)(Yes/No)	22619/53820	25/70	χ2[1]=0.344, p=0.558
Substances taken for depression (drugs or alcohol)(Yes/No)	5131/71308	5/90	χ2[1]=0.129, p=0.720
Talking therapies (Yes/No)	20634/55805	17/78	χ2[1]=3.539, p=0.060
Other non-drug therapies (e.g. yoga) (Yes/No)	8083/68356	5/90	χ2[1]=2.298, p=0.130

Multiple answer questions			
Trouble falling or staying asleep, or sleeping too much (1: not at all-4:nearly every day)	1.91±0.005	1.94±0.138	U=3619934, p=0.988
Recent feelings of inadequacy (1: not at all-4:nearly every day)	1.36±0.004	1.33±0.098	U=3554178.5, p=0.716
Recent trouble concentrating on things (1: not at all-4:nearly every day)	1.30±0.003	1.38±0.092	U=3434322.5, p=0.190
Recent feelings of depression (1: not at all-4:nearly every day)	1.36±0.003	1.40±0.096	U=3385837, p=0.230
Recent poor appetite or overeating (1: not at all-4:nearly every day)	1.40±0.004	1.37±0.106	U=3612522.5, p=0.943
Recent thoughts of suicide or self-harm (1: not at all-4:nearly every day)	1.07±0.002	1.10±0.063	U=3556579, p=0.549
Recent lack of interest or pleasure in doing things (1: not at all-4:nearly every day)	1.31±0.003	1.48±0.118	U=3351972.5, p=0.070
Recent changes in speed/amount of moving or speaking (1: not at all-4:nearly every day)	1.10±0.002	1.13±0.055	U=3530409, p=0.287
Recent feelings of tiredness or low energy (1: not at all-4:nearly every day)	1.82±0.005	2.00±0.135	U=3187494.5, p=0.026
Fraction of day affected during worst episode of depression (1:less than half the day-4:all day long)	2.94±0.005	2.94±0.136	U=1332636, p=0.458

Frequency of depressed days during worst episode of depression (1:less often-3:every day)	2.39±0.003	2.29±0.088	U=1348177.5, p=0.281
Weight change during worst episode of depression (0: stayed about the same or was on a diet, 1:gained weight, 2:lost weight, 3:both gained and lost weight)	0:13555(32%), 1:8175(19%), 2:17686(42%), 3:2935(7%)	0:23(38%), 1:18(30%), 2:16(27%), 3:3(5%)	p=0.046
Duration of worst depression (1:less than a month-6:over two years)	3.16±0.008	3.40±0.229	U=1319400, p=0.232
Impact on normal roles during worst episode of depression (0:not at all-3:a lot)	1.96±0.005	2.06±0.130	U=1392219, p=0.240
Age at first episode of depression (yrs)	36.46±0.076	36.60±2.014	U=1271678.5, p=0.903
Age at last episode of depression (yrs)	49.75±0.066	50.52±2.003	U=1218252.5, p=0.478
Lifetime number of depressed periods	127.43±1.708	136.71±47.624	U=1373136, p=0.834
**Mania**			
Dual answer questions			
Manifestations of mania (more talkative than usual) (Yes/No)	3418/73021	5/90	p=0.618
Manifestations of mania (more restless than usual)(Yes/No)	8175/68264	11/84	χ2[1]=0.013, p=0.910
Manifestations of mania (my thoughts were racing)(Yes/No)	6868/69571	9/86	χ2[1]=0.000, p>0.99
Manifestations of mania (needed less sleep than usual)(Yes/No)	2446/73993	3/92	p>0.99
Manifestations of mania (more creative than usual)(Yes/No)	1629/74810	3/92	p=0.459
Manifestations of mania (was easily distracted)(Yes/No)	6050/70389	9/86	χ2[1]=0.139, p=0.710
Manifestations of mania (was more confident than usual)(Yes/No)	1788/74651	2/93	p>0.99
Manifestations of mania (was more active than usual)(Yes/No)	3560/72879	6/89	p=0.457
Ever had a period of mania/excitability? (Yes/No)	2699(4%)/72286(96%)	8(9%)/86(91%)	p=0.020
Ever had a period of extreme irritability? (Yes/No)	19708/54139	28/64	χ2[1]=0.482, p=0.488
Severity of problems due to mania or irritability (No problems/caused problems)	12387/4893	17/7	χ2[1]=0.000, p>0.99
Multiple answer questions			
Longest period of mania or irritability (1: less than 24hrs-3: a week or more)	1.87±0.006	1.96±0.146	U=226797, p=0.504
**Anxiety**			

Dual answer questions			
Ever felt worried, tense or anxious for most of a month or longer (Yes/No)	21663/48664	29/58	χ2[1]=0.156, p=0.693
Ever worried more than most people would in similar situation (Yes/No)	18547/44811	24/58	χ2[1]=0.000, p>0.99
Stronger worrying (than other people) during period of worst anxiety (Yes/No)	15018/3472	20/6	p=0.614
Worried most days during period of worst anxiety (Yes/No)	23462/2607	30/4	p=0.772
Number of things worried about during worst period of anxiety (One thing/More than one thing)	11030/15252	14/19	χ2[1]=0.000, p>0.99
Difficulty stopping worrying during worst period of anxiety (Yes/No)	25033/1270	31/3	p=0.225
Multiple worries during worst period of anxiety (Yes/No)	20153/5710	22/12	χ2[1]=2.721, p=0.099
Tense, sore or aching muscles during worse period of anxiety (Yes/No)	10714/13835	17/15	χ2[1]=0.814, p=0.367
Difficulty concentrating during worst period of anxiety (Yes/No)	19580/6073	28/7	χ2[1]=0.097, p=0.755
More irritable than usual during worst period of anxiety (Yes/No)	17422/7083	29/4	χ2[1]=3.739, p=0.053
Restless during period of worst anxiety (Yes/No)	14721/10128	24/10	χ2[1]=1.371, p=0.242
Keyed up or on edge during worst period of anxiety (Yes/No)	20731/4966	26/7	χ2[1]=0.003, p=0.957
Frequent trouble falling or staying asleep during worst period of anxiety (Yes/No)	22484/3855	27/7	p=0.329
Easily tired during worst period of anxiety (Yes/No)	19085/6157	26/9	χ2[1]=0.000, p>0.99
Professional informed about anxiety (Yes/No)	16979/9893	19/16	χ2[1]=0.838, p=0.360
Substances taken for anxiety (unprescribed)(Yes/No)	2501(3%)/73938(97%)	7(7%)/88(93%)	p=0.037
Substances taken for anxiety (prescribed)(Yes/No)	11511/64928	13/82	χ2[1]=0.053, p=0.817
Substances taken for anxiety (drugs or alcohol) (Yes/No)	3964/72475	4/91	p>0.99
Activities undertaken to treat anxiety (talking therapies)(Yes/No)	11601/64838	11/84	χ2[1]=0.695, p=0.404
Activities undertaken to treat anxiety (other therapeutic activities e.g. yoga)(Yes/No)	5843/70596	3/92	χ2[1]=2.108, p=0.147
Multiple answer questions			
Recent easy annoyance or irritability (1: not at all-4:nearly every day)	1.34±0.002	1.44±0.061	U=3213617, p=0.018
Recent feelings of nervousness or anxiety (1: not at all-4:nearly every day)	1.41±0.002	1.42±0.073	U=3594330, p=0.913
Recent inability to stop or control worrying (1: not at all-4:nearly every day)	1.37±0.002	1.51±0.087	U=3308660, p=0.113
Recent feelings of foreboding (1: not at all-4:nearly every day)	1.26±0.002	1.34±0.069	U=3340145, p=0.118
Recent trouble relaxing (1: not at all-4:nearly every day)	1.43±0.003	1.52±0.086	U=3477052, p=0.428
Recent restlessness (1: not at all-4:nearly every day)	1.17±0.002	1.25±0.060	U=3380896, p=0.059
Recent worrying too much about different things (1: not at all-4:nearly every day)	1.47±0.003	1.53±0.090	U=3545082, p=0.872
Longest period spent worried or anxious (months)	190±2	164±63	U=289967.5, p=0.471
Frequency of inability to stop worrying during worst period of anxiety (0:Never-3:Often)	2.32±0.004	2.40±0.131	U=428042, p=0.328

Frequency of difficulty controlling worry during worst period of anxiety (0:Never-3:Often)	2.35±0.004	2.49±0.126	U=406215, p=0.138
Impact on normal roles during worst period of anxiety (0: Not at all-3: A lot)	1.84±0.006	2.20±0.152	U=371698, p=0.022
**Addictions**			
Ever addicted to any substance or behaviour (Yes/No)	3820/71829	3/91	p=0.634
Ever addicted to alcohol (Yes/No)	1340/2213	2/1	p=0.561
Ongoing addiction to alcohol (Yes/No)	579/741	1/1	p>0.99
Ever physically dependent on alcohol (Yes/No)	279/997	0/2	p>0.99
Ever addicted to prescription or over-the-counter medication (Yes/No)	670/3093	1/2	p=0.445
Ever addicted to illicit or recreational drugs (Yes/No)	217/3579	0/3	p>0.99
Ever addicted to a behaviour or miscellaneous	643/3145	1/2	p=0.428
Ongoing behavioural or miscellaneous addiction (Yes/No)	327/302	1/0	p>0.99
**Alcohol use**			
Frequency of drinking alcohol (0: Never-4: 4 or more times a week)	2.43±0.005	2.31±0.136	U=3429009.5, p=0.345
Amount of alcohol drunk on a typical drinking day (1: 1-2 units-5: 10 or more units)	1.60±0.003	1.44±0.092	U=2542520, p=0.033
Frequency of consuming 6 or more units of alcohol (1: Never-5: daily or almost daily)	1.69±0.004	1.56±0.102	U=2758770, p=0.304
Frequency of inability to cease drinking in last year (1: Never-5: daily or almost daily)	1.22±0.004	1.06±0.056	U=556594.5, p=0.076
Frequency of failure to fulfil normal expectations due to drinking alcohol in last year (1: Never-5: daily or almost daily)	1.08±0.002	1.03±0.028	U=594290.5, p=0.341
Frequency of needing morning drink of alcohol after heavy drinking session in last year (1: Never-5: daily or almost daily)	1.01±0.001	1.06±0.056	U=604122, p=0.012
Frequency of feeling guilt or remorse after drinking alcohol in last year (1: Never-5: daily or almost daily)	1.33±0.004	1.11±0.053	U=544904, p=0.090
Frequency of memory loss due to drinking alcohol in the last year (1: Never-5: daily or almost daily)	1.17±0.003	1.14±0.099	U=573224, p=0.200
Ever been injured or injured someone else through drinking alcohol (0: No, 1:Yes, but not in last year, 2:Yes, during the last year)	0:74194, 1:1897, 2:302	0:93, 1:2, 2:0	p>0.99
Ever had known person concerned about, or recommend reduction of, alcohol consumption (0: No, 1:Yes, but not in last year, 2:Yes, during the last year)	0:72584, 1:2059, 2:1695	0:90, 1:3, 2:2	p=0.865
Age when known person last commented about drinking habits (yrs)	50.1±0.3	47.3±10.7	U=2607, p=0.908
**Cannabis use**			
Ever taken cannabis (0:No, 1:1-2 times, 2:3-10 times, 3:11-100 times, 4: more than 100 times)	0.35±0.003	0.25±0.062	U=3573919.5, p=0.720
Maximum frequency of taking cannabis (1:Less than once a month, 2:once a month or more but not every week, 3:once a week or more, but not every day, 4:Every day)	1.57±0.008	1.24±0.161	U=93387, p=0.092
Age when last took cannabis (yrs)	31.1±0.1	31.9±3.0	U=115429.5, p=0.744

**Unusual and psychotic experiences**			
Dual answer questions			
Ever seen an unreal vision (Yes/No)	2692/72930	4/90	p=0.579
Ever heard an unreal voice (Yes/No)	1384/74696	2/93	p=0.692
Ever believed in unreal communications or signs (Yes/No)	530/75688	2/93	p=0.142
Ever believed in unreal conspiracy against self (Yes/No)	498/75783	1/94	p=0.464
Ever talked to a health professional about unusual or psychotic experiences (Yes/No)	777/3188	2/4	p=0.335
Ever prescribed medication for unusual or psychotic experiences (Yes/No)	388/3572	2/4	p=0.111
Multiple answer questions			
Number of times seen an unreal vision	31.8±1.4	49.3±46.9	U=3719, p=0.518
Number of times heard an unreal voice	55.7±2.9	103.5±106.5	U=998.5, p=0.831
Number of times believed in unreal communications or signs	62.6±4.5	107.5±102.5	U=239, p=0.279
Number of times believed in unreal conspiracy against self	26.1±1.8	30.0±0.0	U=121.5, p=0.474
Frequency of unusual or psychotic experiences in last year (0:Not at all-4: Nearly every day or daily)	0.42±0.013	0.67±0.494	U=10899.5, p=0.638
Age when first had unusual or psychotic experience (yrs)	34.7±0.4	48.2±10.4	U=7062, p=0.182
Distress caused by unusual or psychotic experiences (0:Not distressing at all-4: Very distressing)	1.41±0.021	1.83±0.749	U=10298.5, p=0.606
**Traumatic events**			
Felt loved as a child (0:Never true-4: Very often true)	3.24±0.004	3.32±0.095	U=3471803, p=0.457
Someone to take to doctor when needed as a child (0:Never true-4: Very often true)	3.73±0.003	3.85±0.043	U=3404766, p=0.243
Been in a confiding relationship as an adult (0:Never true-4: Very often true)	2.98±0.005	2.99±0.138	U=3338756.5, p=0.856
Able to pay rent/mortgage as an adult (0:Never true-4: Very often true)	3.70±0.003	3.61±0.100	U=3424319, p=0.570
Been in serious accident believed to be life-threatening (0:Never, 1:Yes, but not in last 12 months, 2:Yes, within the last 12 months)	0:71274, 1:4882, 2:174	0:83, 1:12, 2:0	p=0.060
Been involved in combat or exposed to war zone (0:Never, 1:Yes, but not in last 12 months, 2:Yes, within the last 12 months)	0:75355, 1:1002, 2:23	0:95, 1:0, 2:0	p=0.650
Diagnosed with life-threatening illness (0:Never, 1:Yes, but not in last 12 months, 2:Yes, within the last 12 months)	0:64635, 1:10067, 2:1371	0:78, 1:16, 2:0	p=0.293
Victim of physically violent crime (0:Never, 1:Yes, but not in last 12 months, 2:Yes, within the last 12 months)	0:65682, 1:10283, 2:283	0:81, 1:13, 2:0	p=0.916
Witnessed sudden violent death (0:Never, 1:Yes, but not in last 12 months, 2:Yes, within the last 12 months)	0:69572, 1:6424, 2:295	0:87, 1:8, 2:0	p>0.99
Avoided activities or situations because of previous stressful experience in past month (0:Not at all-4:Extremely)	0.34±0.003	0.52±0.094	U=3269628, p=0.026
Repeated disturbing thoughts of stressful experience in past month (0:Not at all-4:Extremely)	0.44±0.003	0.53±0.084	U=3361072, p=0.129

Felt very upset when reminded of stressful experience in past month (0:Not at all-4:Extremely)	0.62±0.003	0.76±0.097	U=3308762, p=0.099
Felt irritable or had angry outbursts in past month (0:Not at all-4:Extremely)	0.49±0.004	0.63±0.119	U=951869.5, p=0.217
Felt distant from other people in past month (0:Not at all-4:Extremely)	0.64±0.005	0.78±0.131	U=941187, p=0.179
**Happiness and subjective wellbeing**			
General happiness (1:Extremely happy-6:Extremely unhappy)	2.42±0.003	2.42±0.092	U=3560178, p=0.812
General happiness with own health (1:Extremely happy-6:Extremely unhappy)	2.63±0.003	2.59±0.087	U=3566806.5, p=0.795
Belief that own life is meaningful (1:Not at all-5:an extreme amount)	3.70±0.003	3.70±0.114	U=3317623, p=0.285

Supplementary Table 9. List of medications commonly used to treat heart arrythmia, ADHD-related symptoms and mood symptoms.

Drug type	Effect on symptoms	Biobank Code	Drug
Beta-blocker	Anti-arrhythmia	1140866738	atenolol
	Anti-arrhythmia	1141146126	atenolol+bendrofluazide
	Anti-arrhythmia	1141194810	atenolol+bendroflumethiazide
	Anti-arrhythmia	1141180778	atenolol+chlortalidone
	Anti-arrhythmia	1141146124	atenolol+chlorthalidone
	Anti-arrhythmia	1141146128	atenolol+co-amilozide
	Anti-arrhythmia	1140860426	atenolol+nifedipine 50mg/20mg m/r capsule
	Anti-arrhythmia	1140879760	bisoprolol
	Anti-arrhythmia	1140864950	bisoprolol fumarate+hydrochlorothiazide 10mg/6.25mg tablet
	Anti-arrhythmia	1140879818	metoprolol
	Anti-arrhythmia	1140860308	metoprolol tartrate+chlorthalidone 100mg/12.5mg tablet
	Anti-arrhythmia	1140879854	sotalol
	Anti-arrhythmia	1140860332	sotalol hydrochloride+hydrochlorothiazide 80mg/12.5mg tablet
	Anti-arrhythmia	1140860404	metoprolol tartrate+hydrochlorothiazide 100mg/12.5mg tablet
Calcium channel blocker	Anti-arrhythmia	1140888510	verapamil
	Anti-arrhythmia	1140879806	diltiazem
	Anti-arrhythmia	1140926778	diltiazem hcl+hydrochlorothiazide 150mg/12.5mg m/r capsule
Cardiac glycoside	Anti-arrhythmia	2038459814	digoxin
	Anti-arrhythmia	1140865966	digoxin product
Sodium channel blocker	Anti-arrhythmia	1140888570	flecainide
Calcium and potassium channel blocker	Anti-arrhythmia	1140888502	amiodarone
	Reduction in inattention, hyperactivity and/or impulsive symptoms	1140867894	pemoline
	Reduction in inattention, hyperactivity and/or impulsive symptoms	1140917138	ritalin 10mg tablet
Centrally Acting Sympathomimetics	Reduction in inattention, hyperactivity and/or impulsive symptoms	1141199446	atomoxetine
	Reduction in inattention, hyperactivity and/or impulsive symptoms	1141180976	dexamfetamine
	Reduction in inattention, hyperactivity and/or impulsive symptoms	1140879680	dexamphetamine
Tricyclic Antidepressants	Antidepressant/treatment of mood symptoms	1140867938	amitriptyline+chlordiazepoxide 12.5mg/5mg capsule

Monoamine Oxidase A Inhibitors Reversible	Antidepressant/treatment of mood symptoms	1140867920	moclobemide
Monoamine Oxidase A and B Inhibitors Irreversible	Antidepressant/treatment of mood symptoms	1140867914	tranylcypromine
Selective Serotonin Reuptake Inhibitors	Antidepressant/treatment of mood symptoms	1140867888	paroxetine
Selective Serotonin Reuptake Inhibitors	Antidepressant/treatment of mood symptoms	1140867878	sertraline
Monoamine Oxidase A and B Inhibitors Irreversible	Antidepressant/treatment of mood symptoms	1140867856	isocarboxazid
	Antidepressant/treatment of mood symptoms	1140867852	nardil 15mg tablet
Monoamine Oxidase A and B Inhibitors Irreversible	Antidepressant/treatment of mood symptoms	1140867850	phenelzine
Tricyclic Antidepressants	Antidepressant/treatment of mood symptoms	1140867818	nortriptyline
	Antidepressant/treatment of mood symptoms	1140867812	norval 10mg tablet
Tetracyclic Antidepressants	Antidepressant/treatment of mood symptoms	1140867774	amoxapine
Tricyclic Antidepressants	Antidepressant/treatment of mood symptoms	1140867756	trimipramine
Tricyclic Antidepressants	Antidepressant/treatment of mood symptoms	1140867726	lofepramine
Tricyclic Antidepressants	Antidepressant/treatment of mood symptoms	1140867640	doxepin
Tricyclic Antidepressants	Antidepressant/treatment of mood symptoms	1140867632	dothapax 25mg capsule
Tricyclic Antidepressants	Antidepressant/treatment of mood symptoms	1140867624	prothiaden 25mg capsule
	Antidepressant/treatment of mood symptoms	1140856074	butriptyline
Tricyclic Antidepressants	Antidepressant/treatment of mood symptoms	1140909806	dosulepin
Monoamine Oxidase A and B Inhibitors Irreversible	Antidepressant/treatment of mood symptoms	1140910504	maoi - isocarboxazid
Monoamine Oxidase A and B Inhibitors Irreversible	Antidepressant/treatment of mood symptoms	1140910704	maoi - phenelzine
Monoamine Oxidase A and B Inhibitors Irreversible	Antidepressant/treatment of mood symptoms	1140910820	maoi - tranylcypromine
Serotonin and Noradrenaline Reuptake Inhibitors	Antidepressant/treatment of mood symptoms	1140916282	venlafaxine
	Antidepressant/treatment of mood symptoms	1140917460	nefazodone
Selective Serotonin Reuptake Inhibitors	Antidepressant/treatment of mood symptoms	1140921600	citalopram
Noradrenaline Reuptake Inhibitors	Antidepressant/treatment	1141151978	reboxetine

	of mood symptoms		
Tetracyclic Antidepressants	Antidepressant/treatment of mood symptoms	1141152732	mirtazapine
Serotonin and Noradrenaline Reuptake Inhibitors	Antidepressant/treatment of mood symptoms	1141176854	bupropion
Selective Serotonin Reuptake Inhibitors	Antidepressant/treatment of mood symptoms	1141180212	escitalopram
Serotonin and Noradrenaline Reuptake Inhibitors	Antidepressant/treatment of mood symptoms	1141200564	duloxetine
Serotonin Uptake Inhibitors	Antidepressant/treatment of mood symptoms	1140882244	molipaxin 50mg capsule
Serotonin Receptor Agonists	Antidepressant/treatment of mood symptoms	1140879730	buspirone
Monoamine Oxidase B Inhibitors	Antidepressant/treatment of mood symptoms	1140879668	selegiline
Serotonin Uptake Inhibitors	Antidepressant/treatment of mood symptoms	1140879634	trazodone
Tricyclic Antidepressants	Antidepressant/treatment of mood symptoms	1140879632	protriptyline
Tricyclic Antidepressants	Antidepressant/treatment of mood symptoms	1140879630	imipramine
	Antidepressant/treatment of mood symptoms	1140879628	dothiepin
	Antidepressant/treatment of mood symptoms	1140879624	desipramine
Tricyclic Antidepressants	Antidepressant/treatment of mood symptoms	1140879620	clomipramine
Tricyclic Antidepressants	Antidepressant/treatment of mood symptoms	1140879616	amitriptyline
Tetracyclic Antidepressants	Antidepressant/treatment of mood symptoms	1140879556	mianserin
Selective Serotonin Reuptake Inhibitors	Antidepressant/treatment of mood symptoms	1140879544	fluvoxamine
Selective Serotonin Reuptake Inhibitors	Antidepressant/treatment of mood symptoms	1140879540	fluoxetine
